# Impairment of IGF-1 Signaling and Antioxidant Response Are Associated with Radiation Sensitivity and Mortality

**DOI:** 10.3390/ijms22010451

**Published:** 2021-01-05

**Authors:** Saeed Y. Aghdam, Doreswamy Kenchegowda, Gregory P. Holmes-Hampton, Maria Moroni, Sanchita P. Ghosh

**Affiliations:** Armed Forces Radiobiology Research Institute, Uniformed Services University of the Health Sciences, Bethesda, MD 20889, USA; doreswamy.kenchegowda.ctr@usuhs.edu (D.K.); gregory.holmes-hampton.ctr@usuhs.edu (G.P.H.-H.); maria.moroni@usuhs.edu (M.M.)

**Keywords:** radiation injury, IGF-1, mitochondria, Nrf2, oxidative stress

## Abstract

Following exposure to high doses of ionizing radiation, diverse strains of vertebrate species will manifest varying levels of radiation sensitivity. To understand the inter-strain cellular and molecular mechanisms of radiation sensitivity, two mouse strains with varying radiosensitivity (C3H/HeN, and CD2F1), were exposed to total body irradiation (TBI). Since Insulin-like Growth Factor-1 (IGF-1) signaling pathway is associated with radiosensitivity, we investigated the link between systemic or tissue-specific IGF-1 signaling and radiosensitivity. Adult male C3H/HeN and CD2F1 mice were irradiated using gamma photons at Lethal Dose-70/30 (LD_70/30_), 7.8 and 9.35 Gy doses, respectively. Those mice that survived up to 30 days post-irradiation, were termed the survivors. Mice that were euthanized prior to 30 days post-irradiation due to deteriorated health were termed decedents. The analysis of non-irradiated and irradiated survivor and decedent mice showed that inter-strain radiosensitivity and post-irradiation survival outcomes are associated with activation status of tissue and systemic IGF-1 signaling, nuclear factor erythroid 2–related factor 2 (Nrf2) activation, and the gene expression profile of cardiac mitochondrial energy metabolism pathways. Our findings link radiosensitivity with dysregulation of IGF-1 signaling, and highlight the role of antioxidant gene response and mitochondrial function in radiation sensitivity.

## 1. Introduction

Acute exposure to ionizing radiation originating from nuclear accidents or malevolent and terror-related use of the radioactive material could compromise the survival of individuals primarily via hematopoietic system impairment. The depletion of the hematopoietic stem cells (HSCs) and reduced hematopoiesis results in the Hematopoietic Acute Radiation Syndrome (H-ARS), which is the primary cause of death following exposure to relatively moderate to higher doses of ionizing radiation. However, the hematopoietic system is not the only organ targeted by radiation exposure at hematopoietic doses; diverse cells or tissue types will suffer from either immediate or delayed effects of radiation exposure that can contribute to mortality associated with radiation exposure [1]. The cause of inherent difference in radiosensitivity due to cross-talk at cellular and molecular level among different animal strains is poorly characterized. It is therefore very likely that the effects of the ionizing radiation on organs other than the hematopoietic system could potentially explain the differential radiosensitivity between strains of the same vertebrate species. This claim is supported by the comparison between different animal strains within the same species that manifest differences in radiosensitivity, but possess similar profiles of hematopoietic elements before and following irradiation exposure in the recovery phase [2,3].

The organs and cells, other than the hematopoietic system, that are highly susceptible to radiation injury include the skin, the gastrointestinal (GI) tract, spermatogenic cells, and the cardiovascular system [4]. The effects of radiation on the cardiovascular system have been explored extensively, and it has been shown that radiation causes both acute and latent effects on the cardiovascular system. For instance, nuclear industry workers and nuclear disaster survivors demonstrate relatively higher rates of cardiovascular complications than the general population [5,6]. Furthermore, in cancer patients, radiation therapy regimens increase the risk of associated cardiac complications and mortalities arising from cardiac failure [7,8,9]. The development of suitable agents to mitigate the radiation effects on the cardiovascular system demands a thorough understanding of the involved signaling pathways impacted by radiation. One of the pivotal signaling pathways that modulates the cell or tissue response to radiation injury and is influenced by radiation is insulin-like growth factor-1 (IGF-1) signaling. Structurally, the IGF-1 receptor (IGF-1R) is very similar to the insulin receptor (IR), and shares over 80% homology in its kinase domain β with IR. Both IGF-1R and IR consist of two alpha subunits linked to two beta subunits by disulfide bonds. While the IR regulates the metabolic functions of cells (i.e., glucose metabolism), the IGF-1R mediates a number of the anabolic and mitogenic (i.e., tissue growth-stimulating) effects of the growth hormone (GH) [10]. The IGFs (somatomedins), including the IGF-1 peptide with a molecular weight of approximately 7.6 kDa were initially identified in 1957 by Salmon and Daughaday, following the characterization of their ability to stimulate [35S]-sulfate incorporation into rat cartilage [11]. A major proportion (≈75%) of the circulating IGF-1 ligand in the bloodstream is synthesized and released by the liver via stimulatory effects of the pituitary-derived GH [12]. As such, IGF-1 serves as the primary mediator of the effects of GH in target cells expressing its cognate receptor, the IGF-1R.

The pleotropic effects of IGF-1 signaling are propagated through several downstream pathways including but not limited to the IRS/PI3K/Akt and Grb/Shc/MAPK pathways [13]. The activation of the IRS/PI3K/Akt pathway promotes the phosphorylation of eNOS on the Ser1179 residue, leading to quick production of nitric oxide (NO) that serves important autocrine/paracrine effects in the cardiovascular system, including regulation of vascular tone, prevention of platelet activation, curbing leukocyte adhesion to the endothelium, and myocardial contractility [14]. Cumulative evidence supports the significance of IGF-1R signaling in cardiovascular homeostasis via regulating key cardiac signatures including contractility, metabolism, tissue remodeling and autophagy [15]. Cardiac IGF-1 deficiency is associated with an increased risk of cardiovascular ailments, whereas IGF-1 signaling activation protects the heart from detrimental metabolic stresses and myocardial infarction [15]. In support of the negative effects of radiation on cardiac IGF-1 signaling, we previously showed that in two different strains of minipigs with different radiosensitivity levels, exposure to hematopoietic doses of radiation impaired the cardiac IGF-1 signaling in irradiated decedent animals but not in control and irradiated survivor animals [13,16]. Furthermore, IGF-1 signaling is shown to regulate vascular redox status [17,18] and some key aspects of mitochondrial function. For example, in vitro, IGF-1 signaling plays essential roles in sustaining cellular viability by stimulating mitochondrial biogenesis, dynamics and turnover via regulating the activation of the Nrf2 transcription factor [19,20]. IGF-1 signaling is indirectly involved in the expression of vascular antioxidant response genes and mitigating the effects of the oxidative stress [18].

Altogether, since IGF-1 signaling is an important mediator of cardiovascular homeostasis that is differentially targeted by ionizing radiation in radiosensitive and radioresistant strains, using two different mouse strains with differential levels of radiosensitivity, we asked whether (1) there were any changes in the systemic IGF-1 signaling between control and irradiated animals from both strain; (2) there were any alterations in the activation status of tissue IGF-1 signaling in heart, lung and kidneys from both strains and (3) if irradiation could differentially impact the oxidative stress response and mitochondrial energy metabolism genes in the hearts of both strains.

## 2. Results

### 2.1. Systemic and Cardiac Impairment of IGF-1 and Nrf2 Signaling in Lethal Dose-70/30 (LD_70/30_) Irradiated CD2F1 and C3H/HeN Mice

**Figure 1:**
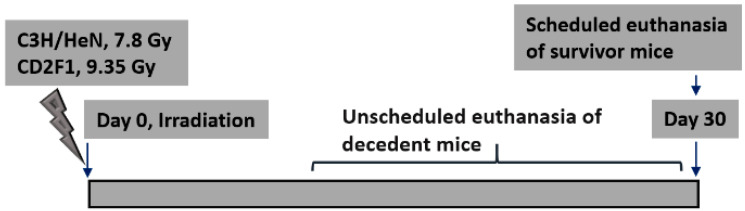
Irradiation scheme for the CD2F1 and C3H/HeN adult male mice. Animals were irradiated at LD_70/30_ dose. The day of irradiation was considered as day 0; animals that underwent unscheduled euthanasia due to severe health deterioration before day 30 post-irradiation were considered as decedents. Animals that survived from radiation exposure were euthanized on day 30 post-irradiation (scheduled euthanasia).

To characterize changes in the systemic and tissue IGF-1 signaling, two mouse strains that manifest disparate radiation sensitivity levels were used in the present study. The CD2F1 strain is comparatively radioresistant, while the C3H/HeN strain radiosensitive [21]. Mice from both strains were irradiated at LD_70/30_ doses, and those mice that survived up to 30 days post-irradiation were euthanized and termed survivors. LD_70/30_ is defined as the dose of radiation expected to cause mortality to 70 percent of exposed mice within 30 days after radiation. Those mice which became moribund and were euthanized before day thirty post-irradiation are referred as decedents (Figure 1).

**Figure 2:**
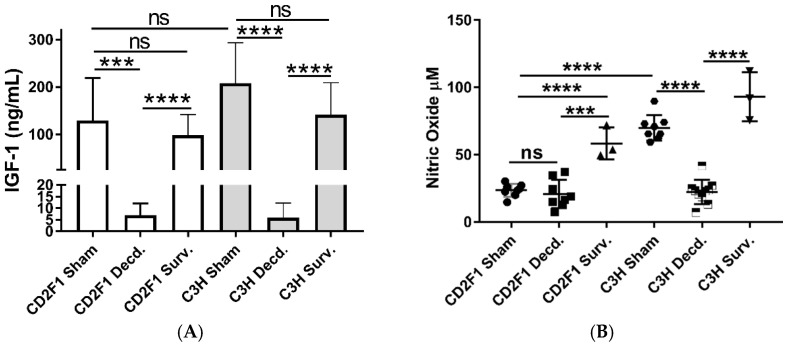
Serum IGF-1 and Nitric Oxide (NO) levels in sham and LD_70/30_ irradiated CD2F1 and C3H/HeN adult male mice. (**A**) ELISA analysis of serum IGF-1 levels in sham, irradiated decedent (Decd.) and irradiated survivor (Surv.) mice. *n* = 7 for sham animals of both strains, *n* = 8 for decedent animals of both strains, *n* = 3 for survivors of both strains. (**B**) Serum nitric oxide levels in sham, irradiated decedent (Decd.) and irradiated survivor (Surv.) mice. *n* = 8 for sham animals of both strains, *n* = 8 for CD2F1 decedents, *n* = 10 for C3H/HeN decedents, *n* = 3 for survivors of both strains. Data analyzed by student’s *t*-test. Results presented as mean ± SEM, *** *p* < 0.001 and **** *p* < 0.0001, *p* > 0.05 considered as ‘not significant’ (ns).

Using minipig models of radiation sensitivity, we previously showed that moribundity associated with hematopoietic doses of radiation was accompanied with changes in serum IGF-1 levels [16]. Therefore, to determine the effects of radiation-associated moribundity in IGF-1 signaling, the serum IGF-1 levels in irradiated (survivors and decedents) and non-irradiated (sham) CD2F1 and C3H/HeN mice were assessed using ELISA. Analysis of serum samples showed reduced levels of IGF-1 ligand in irradiated decedent animals of both strains compared with sham and irradiated survivors (Figure 2A). There were no significant differences in IGF-1 levels between sham animals and irradiated survivors of the respective strains. These findings shows the link between moribundity following radiation exposure at hematopoietic doses and decline in serum IGF-1 levels.

Since nitric oxide (NO) is an important regulator of cardiovascular functions, and its synthesis is impacted by exposure to radiation and modulated by IGF-1 signaling [13,16,22,23], we next tested the serum NO levels in sham and irradiated survivors and decedents of both CD2F1 and C3H/HeN strains. The sham CD2F1 strain had significantly lower levels of basal NO when compared to C3H/HeN sham animals. While decedents of C3H/HeN strain showed significant decline in NO compared with sham C3H/HeN mice, no changes were observed in NO levels between sham and decedent CD2F1 mice (Figure 2B). The survivors of both strains yielded significantly higher NO levels when compared to decedents animals of their respective strains. (Figure 2B).

**Figure 3:**
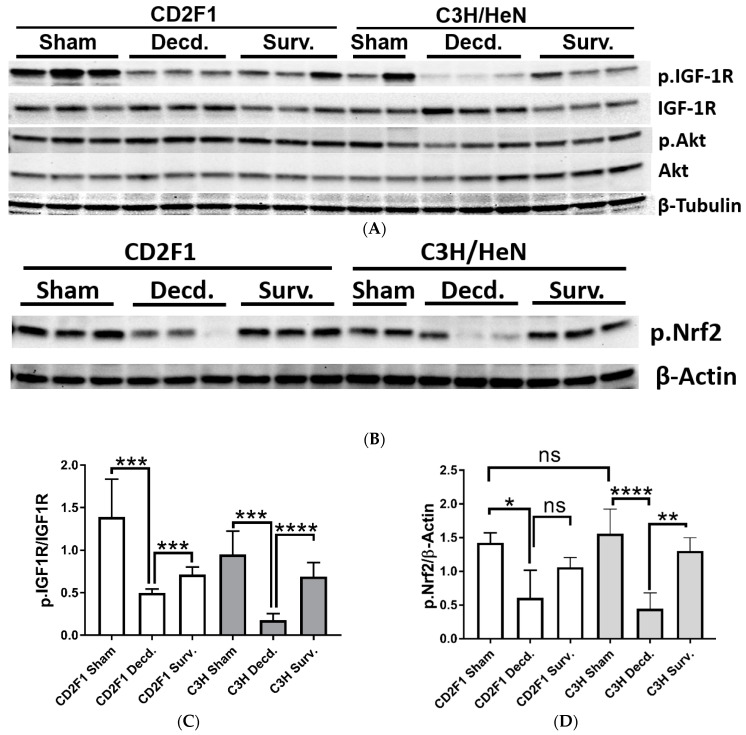
Western blot analysis of IGF-1R, Akt and Nrf2 activation in sham and LD_70/30_ irradiated CD2F1 and C3H/HeN adult male mice. (**A**,**B**) Analysis of phosphorylated IGF-1 receptor (p.IGF1R, Tyr1135/1136), total IGF-1R, phosphorylated Akt (p.Akt, Ser473), total Akt, phosphorylated Nrf2 (p.Nrf2, Ser40 residue), β–Tubulin and β–Actin in heart protein extracts from sham, irradiated decedent (Decd.) and irradiated survivor (Surv.) mice. (**C**,**D**) Quantification of the IGF-1R and Nrf2 activation. The Y axis in graphs represents the ratios of the respective measured pixel intensities of Western blot bands. Total number of analyzed samples from both CD2F1 and C3H strains: sham—*n* = 6; decedent—*n* = 6; survivor *n* = 3. Data analyzed by Student’s *t*-test and are presented as mean ± SEM, * *p* < 0.05, ** *p* < 0.01, *** *p* < 0.001 and **** *p* < 0.0001, *p* > 0.05 considered as ‘not significant’ (ns).

Changes in systemic IGF-1 and NO levels in decedent mice suggested that tissue IGF-1 signaling could be impacted by radiation-induced moribundity. Since both IGF-1 and NO signaling are important mediators of cardiovascular homeostasis [24,25], using SDS-PAGE/Western blot analysis, the activity of IGF-1 signaling in heart tissues of sham, irradiated survivor and decedent CD2F1 and C3H/HeN mice was evaluated. To accomplish this, the phosphorylation of the IGF-1 receptor (IGF-1R, Tyr1135/1136) that is triggered by IGF-1 ligand binding and reflects the activation of the IGF-1 signaling pathway was investigated [26]. IGF-1R activation was evaluated by calculating the ratio of the Western blot band pixel intensities for phosphorylated IGF-1R (Tyr1135/1136) to that of total IGF-1R. IGF-1R activation was diminished in decedent animals from both the strains when compared with respective sham and survivor animals (Figure 3A,C). The inactivation of IGF-1R in decedent animals was more robust in the C3H/HeN strain than the CD2F1 strain since in decedent C3H/HeN hearts, the IGF-1R phosphorylation was almost completely abrogated and the expression of the IGF-1R was elevated. In contrast CD2F1 strain decedents showed mild increase in IGF-1R expression and lesser reduction of IGF-1R activation (Figure 3A,C). The analysis of the IGF-1 downstream signaling via testing the Akt phosphorylation on Ser473 residue showed no significant differences in the activity of Akt in the irradiated decedent animals of both CD2F1 and C3H/HeN strains (Figure 3A).

To determine whether perturbation of cardiac IGF-1 signaling in the irradiated decedent animals was accompanied by changes in Nrf2 activity, Western blot analysis of Nrf2 activation using an antibody that detects the activated form of Nrf2 transcription factor was used (p.Nrf2 Ser40). The phosphorylation of Nrf2 on Ser40 by protein kinase C stimulates the Nrf2 dissociation from its sequestering protein, Keap1 leading to Nrf2 translocation into nucleus and initiation of the transcription of antioxidant response genes [27]. Western analysis of Nrf2 showed significant reduction in Nrf2 activation in irradiated decedent animals of both CD2F1 and C3H/HeN strains (Figure 3B,D). The Nrf2 inactivation was more pronounced in the C3H/HeN decedent mice compared with the CD2F1 decedents (Figure 3B,D).

**Figure 4:**
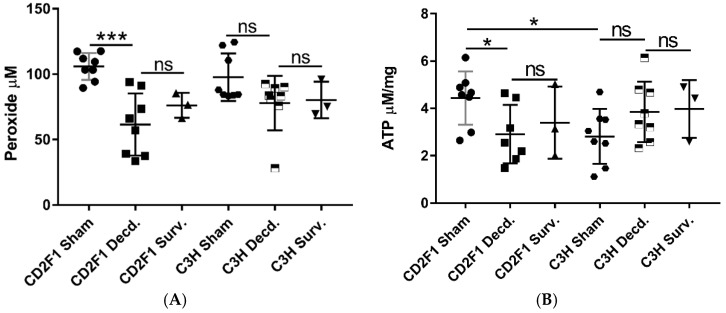
Peroxide and ATP levels in sham and LD_70/30_ irradiated survivor and decedent heart samples from CD2F1 and C3H/HeN mice. Graphs representing the heart peroxide (**A**) and ATP (**B**) levels measured in sham and irradiated CD2F1 and C3H/HeN mice. Total number of analyzed samples: *n* = 8 for sham and irradiated decedent mice and *n* = 3 for irradiated survivors. Data analyzed by Student’s *t*-test, presented as mean ± SEM, * *p* < 0.05, *** *p* < 0.001 and *p* > 0.05, ns—not significant.

Hydrogen peroxide (H_2_O_2_) is one of the extensively studied reactive oxygen species (ROS). Tightly controlled production of H_2_O_2_ plays an important role as a second messenger in various physiological processes. Uncontrolled production of H_2_O_2_ results in oxidative stress, contributes various pathological conditions including cardiovascular diseases through its reaction with other reactive species such as hydroxyl radical and hypochlorous acid. [28]. After we showed reduced cardiac Nrf2 activation in decedent mice, it was asked whether cardiac peroxide levels were affected by changes in Nrf2 activity. Measurement of cardiac peroxide in heart samples of the sham, irradiated survivor and decedent mice revealed that decedent CD2F1 mice had diminished peroxide levels compared with the sham animals (Figure 4A). No statistically significant changes in peroxide levels were observed between sham and decedent C3H/HeN mice. Likewise, no changes in peroxide levels were discernible between sham and survivor animals of each strain (Figure 4A).

Since mitochondria are profoundly susceptible to radiation injury [29] and owing to the involvement of IGF-1 signaling in mitochondrial biogenesis, mitophagy and turnover [19,20], we tested whether the reduced cardiac ATP levels in CD2F1 as a result of impaired IGF-1 signaling or mitochondrial function were directly influenced by irradiation. The analysis of heart samples showed significant reduction of ATP levels in the decedent CD2F1 strain compared with sham animals (Figure 4B). Furthermore, the basal ATP levels in CD2F1 mice were higher compared with C3H/HeN basal levels. (Figure 4B). There were no statistically significant differences in ATP content between sham and irradiated C3H/HeN heart samples (Figure 4B).

### 2.2. Radiation Has Differential Effects on the Expression of Cardiac Genes Involved in Oxidative Stress and Mitochondrial Energy Metabolism in the LD_70/30_ Irradiated CD2F1 and C3H/HeN Mice

Effect of hematopoietic doses of radiation on cardiac IGF-1, Nrf2 and systemic NO signaling, prompted us to test the expression of the genes involved in nitric oxide, oxidative stress, and mitochondrial energy metabolism. We analyzed the expression of the respective genes in the hearts of the sham, irradiated survivor and decedent mice using quantitative PCR array plates. For PCR array analysis, only those genes in irradiated animals that showed three fold or higher differences in their expression when compared with the sham animals are shown.

**Figure 5:**
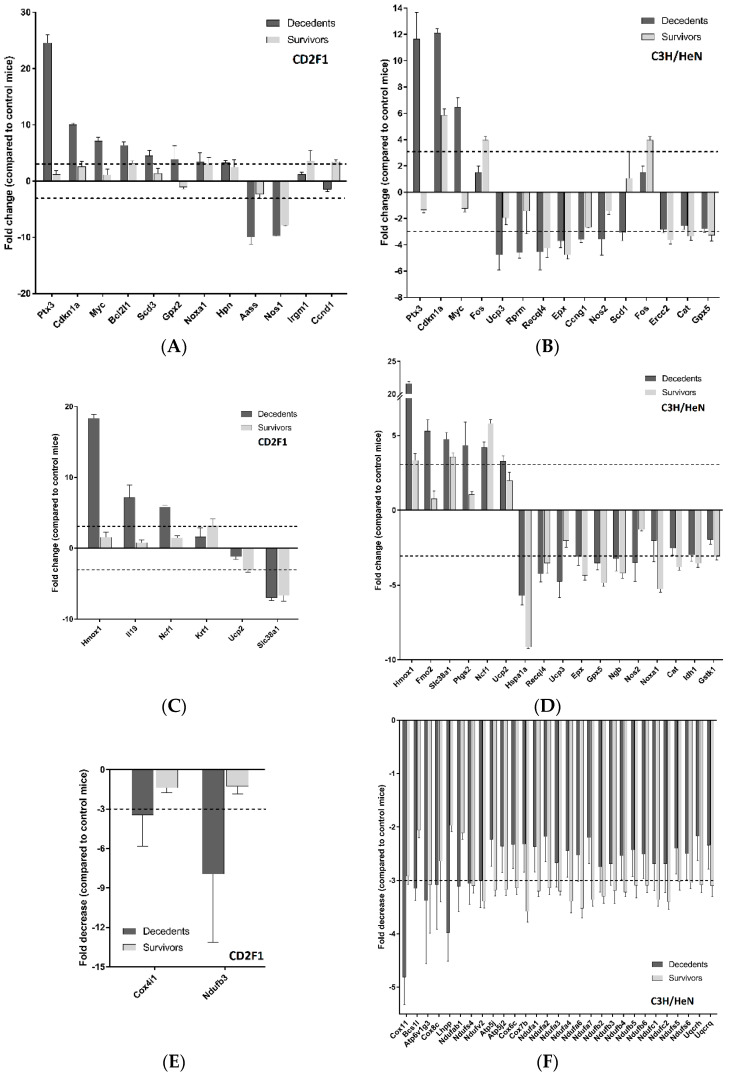
PCR array analysis of genes involved in nitric oxide (NO), oxidative stress and mitochondrial energy metabolism in heart samples from sham and LD_70/30_ irradiated CD2F1 and C3H/HeN mice. Quantitative PCR array analysis showing the fold changes in the expression of NO (**A**,**B**) oxidative stress (**C**,**D**) and mitochondrial energy metabolism (**E**,**F**) mRNA levels in heart tissues from sham, irradiated decedent and irradiated survivor mice. The Y axis shows the relative fold changes in expression of the genes in irradiated survivors and decedents compared with the sham. Only those genes showing changes of three-fold or higher, either in the survivors or decedents are shown. The dotted line indicates 3-fold upregulation or downregulation in the graphs. Number of samples for both CD2F1 and C3H strains: Sham (control)—*n* = 7; decedent—*n* = 8; survivors—*n* = 3.

Gene expression analysis of cardiac genes involved in nitric oxide signaling showed that twelve genes in CD2F1 and fifteen genes in C3H/HeN mice were either up-regulated or down-regulated. Among those genes, three of them, Pentraxin 3 (Ptx3), Cyclin Dependent Kinase inhibitor 1A (Cdkn1a) and the proto-oncogene Myc, were common among both the strains and indeed all three genes showed the highest level of dysregulation in both strains (Figure 5A,B). The analysis of genes involved in oxidative stress signaling in heart revealed that the expression of six genes in CD2F1 strain and seventeen genes in C3H/HeN strain were altered in the irradiated survivors or decedents mice (Figure 5C,D). Among the identified genes, four of them, Heme oxygenase 1 (Hmox1), Neutrophil cytosol factor 1 (Ncf1), Mitochondrial uncoupling protein 2 (Ucp2) and Sodium-coupled neutral amino acid transporter 1 (Slc38a1) were common in both CD2F1 and C3H/HeN strains (Figure 5C,D).

The mitochondrial energy metabolism genes in the heart samples from both CD2F1 and C3H/HeN strains were downregulated by more than three-fold following radiation. According to PCR array results, two genes in CD2F1 strain and twenty-nine genes in C3H/HeN strain were downregulated after irradiation in the decedents or survivors (Figure 5E,F), among which, NADH:Ubiquinone Oxidoreductase Subunit B3 (Ndufb3) was common across both strains (Figure 5E,F).

### 2.3. Radiation Disrupts the IGF-1 Signaling in the Lungs and Kidneys of the Irradiated Decedent C3H/HeN Strain and Inactivates the Nrf2 Signaling in the Lungs of Irradiated Decedent CD2F1 Mice

**Figure 6:**
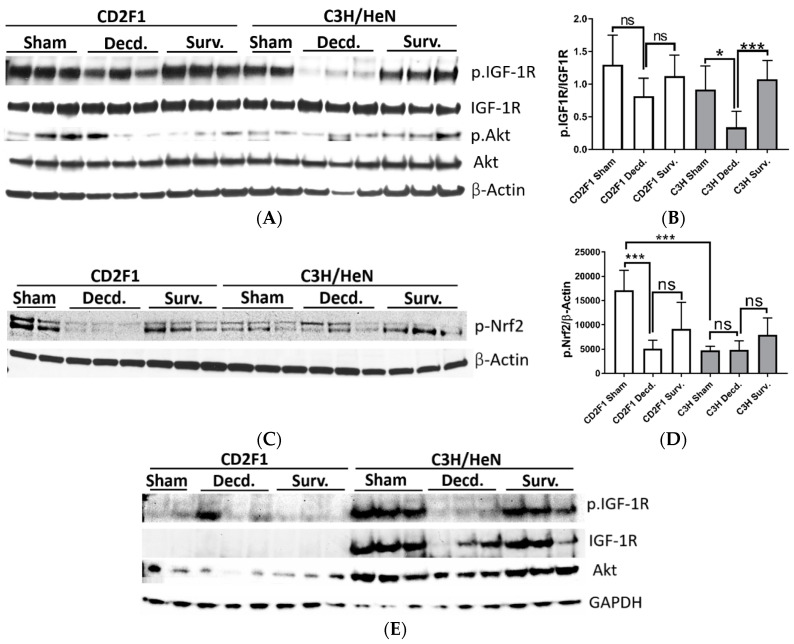
Western blot analysis of IGF-1R, Akt and Nrf2 in lung and kidney samples from sham and LD_70/30_ irradiated CD2F1 and C3H/HeN adult male mice. (**A**) Analysis of phosphorylated IGF-1 receptor (p.IGF1R, Tyr1135/1136), total IGF-1R, phosphorylated Akt (p.Akt, Ser473), total Akt and (**C**) phosphorylated Nrf2 (p.Nrf2, Ser40) in lung protein extracts from sham, irradiated decedent (Decd.) and irradiated survivor (Surv.) mice. β–Actin used as loading control. (**B**,**D**) Quantification of IGF-1R and Nrf2 activation in the lung samples. IGF-1R activation is determined by calculating the ratio of the pixel intensity of p.IGF-1R to total IGF-1R levels. Nrf2 activation is determined by calculating the ratio of pixel intensity of the p.Nrf2 to respective β–Actin band. (**E**) Western blot analysis of phosphorylated IGF-1 receptor (p.IGF1R, Tyr1135/1136), total IGF-1R and total Akt in kidney protein extracts from sham, irradiated decedent (Decd.) and irradiated survivor (Surv.) mice. GAPDH is used as loading control. Total number of analyzed samples for both strains: *n* = 6 for sham, *n* = 6 for decedents, *n* = 3 for survivors. Results analyzed by Student’s *t*-test; presented as mean ± SEM, * *p* < 0.05, *** *p* < 0.001 and *p* > 0.05 is considered as ‘not significant’ (ns).

After showing the systemic and cardiac effects of radiation exposure on IGF-1 and Nrf2 signaling pathways, it was asked whether the IGF-1 and Nrf2 signaling in tissues other than the heart were similarly affected by radiation exposure. To address this question, the activation of IGF-1 and Nrf2 signaling pathways were assessed in the lung and kidneys of the sham and irradiated CD2F1 and C3H/HeN mice using the Western blot analysis and the antibodies that were described in Section 2.1. The analysis of IGF-1 signaling in lungs revealed that the activation of IGF-1R was attenuated in irradiated decedent C3H/HeN mice, as shown by reduced phosphorylation, despite increased total-IGF-1R expression compared with sham (Figure 6A,B). In decedent CD2F1 mice, there was a slight but not statistically significant reduction in IGF-1R receptor phosphorylation, albeit with no apparent changes in total IGF-1R expression in decedent mice compared with the sham and irradiated survivor CD2F1 mice (Figure 6A,B). Furthermore, the phosphorylation of Akt on Ser473 residue and expression of total-Akt were not affected irrespective of the radiation status or strain (Figure 6A).

The analysis of Nrf2 activation in lung samples showed overall weaker Nrf2 activation in sham C3H/HeN mice compared with sham CD2F1 mice (Figure 6C,D). In the lungs from CD2F1 decedents, the Nrf2 activity was diminished significantly compared with the CD2F1 sham animals (Figure 6C,D). In irradiated survivor CD2F1 mice, the Nrf2 activation appeared to be restored when compared with decedent CD2F1; however, the results were statistically insignificant. There were no quantitatively discernable differences in Nrf2 activation between sham, irradiated decedent and survivor C3H/HeN mice (Figure 6C,D).

The Western blot analysis of IGF-1 signaling in kidney samples of sham and irradiated CD2F1 and C3H/HeN mice revealed a pattern consistent with inactivation of IGF-1 signaling in decedent C3H/HeN mice only (Figure 6E). Sham and irradiated CD2F1 mice showed relatively lower IGF-1R expression (Figure 6E). In contrast, the sham C3H/HeN mice had robust expression of IGF-1R that was attenuated in the decedents and restored in survivor animals (Figure 6E). The Western blot analysis of Akt (p.Ser473) did not yield detectable signal in the kidney samples (data not shown), however, similar to IGF-1R expression status, the C3H/HeN mice manifested comparatively higher expression of total Akt protein in their kidneys than CD2F1 strain mice (Figure 6E).

## 3. Discussion

As an essential signal transduction mechanism mediating physical growth in vertebrates, the IGF-1 signaling is also crucial for cardiovascular homeostasis, and its engagement in myriad cardiac functions such as contractility, tissue remodeling, metabolism and autophagy has been documented [15]. Perturbation of the IGF-1 signaling pathway has been associated with a diverse set of diseases, including muscle, cardiovascular, metabolic, neurodegenerative and cancer [30]. According to our previous in vivo findings obtained from comparison of two radiosensitive and radioresistant minipigs strains, the quality and efficiency of cardiac IGF-1 signaling is also tightly associated with total body radiation (TBI) resistance and post-irradiation survival outcomes [13,16], an observation showing consistency with cardioprotective effects of IGF-1 signaling.

Given the established role of IGF-1 signaling in radiation sensitivity [31], in the current report we explored the possibility of whether the link between tissue IGF-1 signaling and radiation sensitivity could be extrapolated to other animal models and if the quality and maintenance of normal IGF-1 signaling in tissues other than heart would show similarities to heart tissue. At a molecular level, we also explored the link between cardiac mitochondrial energy metabolism, cardiac antioxidant gene response mediated by Nrf2 transcription factor and their association with the radiation sensitivity.

Using two mouse strains, CD2F1 and C3H/HeN, that manifest differences in TBI sensitivities [21], we confirmed that radiation exposure in hematopoietic doses not only impaired the IGF-1 signaling in the heart, but also perturbed systemic as well as lung and kidney IGF-1 signaling pathways. In particular, our results reveal striking similarities in the pattern of IGF-1 signaling impairment in the heart, lung and kidney from irradiated C3H/HeN strain and in the hearts from irradiated decedent CD2F1 mice. Considering that serum IGF-1 levels declined in all irradiated decedent mice from both strains and three tissues in C3H/HeN, i.e., the heart, lung and kidney, although only the heart of CD2F1 irradiated decedent mice showed impaired IGF-1R activation, raises the possibility that the radiosensitive C3H/HeN strain is more susceptible to the failure of the molecular components responsible for safeguarding or propagating the IGF-1 signaling. This finding is supported by virtually complete absence of IGF-1R phosphorylation in the heart, lung and kidney samples from irradiated decedent C3H/HeN mice but not in CD2F1 mice. Contrarily, IGF-1 signaling is apparently steadfast in the C3H/HeN strain since compared with the wild type C57BL/6J strain: the sera from C3H/HeN strain mice manifest 35% higher IGF-1 levels [32]. In agreement with higher than wild type serum levels of IGF-1 in C3H/HeN, the C3H/HeN mice have significantly larger femoral and cortical area, both of which are associated with the strength of the GH/IGF-1 signaling [11,32]. Serum IGF-1 levels between sham CD2F1 and C3H/HeN mice were not altered presumably due to the autocrine and paracrine effects of IGF-1. Further, the differences in IGF-1 binding proteins, or other intracellular mediators of IGF-1 signaling such as protein phosphatases (i.e., PTP1B, PTEN) and also microRNA regulate the levels of circulating IGF-1 and its activity [30,33,34].The underlying cause for post-irradiation impairment of tissue IGF-1 signaling as seen in the heart, lung and kidney samples of the CD2F1 and C3H/HeN mice requires further investigation, but could be attributed to the ROS content and the redox status of the tissues [34,35]. It is known that ROS, which drives oxidative stress in biological systems, has inhibitory effects on insulin and IGF-1 signaling [36,37], and IGF-1 signaling itself suppresses the oxidative stress in vivo in vasculature and in vitro in endothelial cells [17]. Indeed, the reduction in serum NO levels in decedent mice from both strains combined with PCR array data from heart tissue shows inter-strain differences in the expression of oxidative stress response genes. Further, alterations in the activity Nrf2 in the heart and lung tissues of irradiated CD2F1 and C3H/HeN mice compared to sham suggests the involvement of oxidative stress in the dysregulation of IGF-1 signaling. The synthesis of NO by the enzyme, endothelial-nitric oxide synthases (eNOS), requires tetrahydrobiopterin (BH4) that itself is a major target for oxidation by peroxynitrite (ONOO^−^) [38]. Peroxynitrite is formed as a result of NO reaction with superoxide radicals, and reduction in BH4 levels will lead to reduced NOS activity or its uncoupling and enhanced peroxide production, oxidative stress and vascular dysfunction [39]. Thus, relatively higher rates of change in the expression of the genes involved in oxidative stress response and nitric oxide signaling pathways in C3H/HeN mice compared with CD2F1 mice hints at the existence of higher levels of oxidative stress and vascular distress in C3H/HeN mice compared with CD2F1 mice. Likewise, declined NO levels in decedents of both CD2F1 and C3H/HeN mouse strains is similar to our previous findings in minipigs models of ARS where decedent radioresistant and radiosensitive minipigs strains manifested declining NO in the their sera that was accompanied with cardiac IGF-1 signaling impairment and enhanced oxidative stress [13,16].

Our data on differential mitochondrial gene expression changes observed in the heart samples from CD2F1 and C3H/HeN mice provide important clues on the possible involvement of the mitochondria in radiation sensitivity, ROS generation, and possibly the observed dysregulated IGF-1 signaling in irradiated decedent mice. Scientific evidence has shown that mitochondria are among the primary cellular targets of radiation injury, by virtue of their relatively large size and higher proportional abundance among other organelles. For instance, mitochondria may occupy up to one-third of the volume of cardiomyocytes in healthy adult heart [40]. The post-irradiation impairment of the mitochondrial physiology contributes to perturbation of oxidation-reduction reactions that govern the cellular redox status [41]. The measurement of ATP in heart samples of sham and irradiated CD2F1 and C3H/HeN mice revealed no striking differences, except in sham CD2F1 mice that produce higher ATP levels than sham C3H/HeN hearts. The abundant number of the mitochondrial gene expression changes in hearts of irradiated C3H/HeN compared with irradiated CD2F1 strain, clearly supports the major impairment of mitochondrial physiology in C3H/HeN mice. The reported regulation of some of the key mitochondrial features by IGF-1 signaling [19,20], the correlation of differential regulation of IGF-1 signaling, and differential mitochondrial energy metabolism and oxidative stress gene regulations in the hearts of CD2F1 and C3H/HeN supports the hypothesis that redox status might determine the activity or efficiency of the IGF-1 signaling pathway in mice.

Collectively, these data suggest that exposure of radiosensitive and radioresistant mouse strains to hematopoietic doses of radiation results in declined systemic IGF-1 levels and tissue IGF-1 signaling in moribund animals. The impairment of tissue IGF-1 signaling in the radioresistant CD2F1 strain is restricted only to cardiac tissue out of three tissues studied here (heart, lung, and kidney). However, in the irradiated radiosensitive C3H/HeN strain the impairment of IGF-1 signaling was observed in all three examined tissues. Furthermore, in cardiac tissue, the extent of IGF-1 signaling impairment was more pronounced in the radiosensitive strain than in radioresistant strain. The subsequent Nrf2 signaling and qPCR array analysis also revealed the effect of radiation on oxidative stress response and mitochondrial energy metabolism systems of the cardiac tissue in both strains. Interestingly, the PCR array revealed that the intensity of perturbations in oxidative stress and mitochondrial energy metabolism genes was greater in radiosensitive strain than in radioresistant strain. In conclusion, the mechanisms governing radiation sensitivity are likely complex and represent the sum effects of radiation on mitochondrial function, redox status of the cells and the activation status of IGF-1 in different tissues. Thus, regardless of the complexity of signaling and molecular relay nexuses involved in radiosensitivity, inactivation of systemic and tissue IGF-1 signaling, together with the failure of the antioxidant response system, are major mechanisms underlying radiosensitivity leading to radiation-associated moribundity.

## 4. Materials and Methods

### 4.1. Animal Strains, Radiation, Blood and Tissue Collection from Animals

For total body irradiation (TBI), 36 adult 14–16 weeks old male CD2F1 and 36 C3H/HeN male mice (both sourced from Envigo; Indianapolis, IN, USA) were irradiated bilaterally at the Armed Forces Radiobiology Research Institute (AFRRI, Bethesda, MD, USA) using ^60^Co gamma irradiation (~0.6 Gy/min, midline dose) at 9.35 Gy and 7.8 Gy respectively. 9.35 Gy and 7.8 Gy represent the LD_70/30_ radiation doses for each strain. During irradiation, the animals were placed in breathable Plexiglas^®^ chambers specifically fabricated for mouse irradiation. Sham animals were loaded into Plexiglas^®^ chambers but did not receive any radiation doses during the duration of the irradiation. The mice that survived until the endpoint date of the study, day 30, were euthanized and termed survivors. Those irradiated mice who manifested irradiation-associated severe health deteriorations and manifested poor survival prognosis according to approved health-related endpoints, were euthanized before day 30 and termed decedents. Sixteen adult (14–16 weeks old) male mice of both CD2F1 and C3H/HeN strains were included in the experiments as sham non-irradiated animals. During the experimental procedures, the mice were handled in accordance with the specific approval from the institutional animal care and use committee (IACUC) and the Guide for the Care and Use of Laboratory Animals, animal protocol number: P-2018-02-001 at the AFRRI, Veterinary Science Department (Bethesda, MD, USA). The mice were housed in approved cages in counts of 4–5 mice per cage and maintained on a 12 h light–dark cycle in rooms set at 61–81 Fahrenheit with 30–70% relative humidity. At various time points, i.e., day 30 for irradiated survivors and sham animals and also in unscheduled euthanasia times for decedent animals, blood was collected via cardiocentesis under isoflurane anesthesia. For serum isolation, the blood was collected in BD microtainer tubes (Beckton, Dickinson and Company, Franklin Lakes, NJ, USA), allowed to clot 2–3 h at room temperature and serum was separated via centrifugation at 2400× *g* for 10 min. Serum was immediately frozen in 20–50 µL aliquots on dry ice and stored at −80 °C until used. Following blood collection, the mice were euthanized by cervical dislocation and transcardially perfused with 10 mL of Phosphate-buffered saline (PBS) buffer. The lungs, hearts, and kidneys were collected and flash frozen in liquid nitrogen and stored in −80 °C until processed.

### 4.2. Western Blot Analysis

For protein extraction, the frozen tissues were pulverized using Bessman tissue pulverizer (Thomas Scientific, Swedesboro, NJ, USA) on dry ice and 30 mg of frozen powder was homogenized in 300 µL of ice-cold Radioimmunoprecipitation assay (RIPA) buffer (Thermo Fisher, Waltham, MA, USA) supplemented with protease and phosphatase inhibitors. The protein concentration in homogenates was measured using Pierce BCA protein assay kit (Thermo Fisher). Equal amounts of chemically reduced protein lysates were separated using 4–15% Criterion™ gradient polyacrylamide gels and transferred onto PVDF sheets (both from BioRad, Hercules, CA, USA). Blocking and incubation with antibodies were carried out using Western Breeze Chemiluminescent kit (Thermo Fisher). Antibodies: AKT (9272), p.AKT (9271), IGF-1R (9750), p.IGF-1R (3024), β-Actin (4970), β-Tubulin (2128), GAPDH (2118) (Cell Signaling); pSer40.Nrf2 (LS-C497655) (LSBIO, Seattle, WA, USA) were diluted in the vendor recommended dilutions in blocking solution and incubated overnight by rocking in 4 °C. Novex^®^ AP Chemiluminescent Substrate (Thermo Fisher) was used to develop the blots and densitometric analysis of proteins was performed using ChemiDoc system and Image Lab 5.2.1 Software (BioRad) or ImageJ (NIH, Bethesda, MD, USA). Where stated, the β-Actin/Tubulin and GAPDH blots were used for normalization of target bands as the loading control for Western blot analyses. For each group of animals the protein lysates from the tissues of at least 6 random animals (for sham and irradiated decedents) and 3 individual irradiated survivor animals were analyzed three times or more by Western blot technique.

### 4.3. ELISA, Peroxide, ATP and Nitric Oxide Measurements

Frozen serum samples were thawed on ice and subsequently diluted 1:100 times to measure the concentration of IGF-1 using the mouse IGF-1 ELISA Kit PicoKine™ (Boster Bio, Pleasanton, CA, USA) by following the vendor instructions.

For tissue peroxide measurements, 25 mg of frozen heart powder was homogenized in 300 µL of lysis buffer containing 0.1 M KCl and 0.1 M Na_2_HPO_4_·7H_2_O. Following mechanical homogenization, the lysates were spun at 14,000× *g* for 15 min at 4 °C and the supernatant was used for peroxide measurement using the Pierce Quantitative Peroxide Assay Kit (Thermo Fisher) according to kit instructions.

To measure the heart ATP levels, 20 mg of frozen heart powder was homogenized in Tris-EDTA-saturated phenol (phenol-TE) using hand-held tissue homogenizer [42]. Following spinning at 14,000× *g*, the supernatant was extracted twice by adding 400 µL of chloroform. The supernatant was diluted 1/50 in water and ATP levels were measured using ATP Determination Kit (Thermo Fisher).

For nitric oxide measurement, 150 µl of each serum sample was clarified using Amicon-10 kDa spin filters (MilliporeSigma, Burlington, MA, USA) by centrifugation at 14,000× *g* for 1 h at 4 °C. 50 µL of the filtrate was assayed in each well of a 96-well microplate according to total nitric oxide and nitrate/nitrite parameter assay kit (R&D Systems, Minneapolis, MN, USA). Briefly, first the nitrate in samples was enzymatically converted to nitrite by nitrate reductase. Subsequently, the concentration of the nitrite is determined by colorimetric Griess Reaction that is determined by plotting unknown sample values against a nitrite standard curve (1–10 µM range). The plates for the above experiments were read using Spectramax 250 plate reader (Molecular Devices, San Jose, CA, USA) according to recommended settings.

### 4.4. Real-Time PCR Array Analysis

For quantitative analysis of gene expression in the mouse heart samples, the pulverized frozen heart samples were transferred into a 1.5 mL Eppendorf tubes containing 800 µL of QIAzol lysis reagent (Qiagen, Hilden, Germany) pre-warmed to 37 °C. The mix was vigorously vortexed at maximum speed for 30 s. Samples were incubated for five minutes at room temperature (RT), vortexing frequently followed by adding 0.2 mL of chloroform to the QIAzol, and vortexing for additional 15 s. Tubes were spun at 15,000× *g* for 10 min and the top layer was transferred to a new tube containing 700 µL of Qiagen RLT buffer. Subsequently 500 µL of 96–100% ethanol was added to each tube and vortexed for another 15 s. The cocktail was applied into individual RNeasy plus universal mini kit columns (Qiagen) and RNA samples were purified by following the manufacturer’s instructions. The quantitative and qualitative assessment of the RNA samples was performed using NanoDrop C (Thermo Fisher) and 3 µg of total RNA was used for cDNA synthesis. For cDNA synthesis, random hexamers and the SuperScript™ IV First-Strand Synthesis System (Thermo Fisher) were used by following the recommended steps and procedure in a standard thermocycler machine (Thermo Fisher). For Real-time PCR analysis using RT2 profiler arrays (Qiagen), 12.5 µL of 2X SABioscience SYBR Green master mix (Qiagen), 1 µL of synthesized cDNA and 11.5 µL H_2_O were mixed and added into each well of a PCR array plate and assayed in QuantStudio3 Real-time PCR machine (Thermo Fisher). To calculate the changes in the transcript numbers for any target gene, the CT (cycle threshold) values were entered in the online Qiagen gene array expression analysis tool. Changes in the fold change for every individual gene in irradiated survivor or decedent animals were compared to the CT values obtained from non-irradiated sham animals.

### 4.5. Statistical Analysis

Data analysis was performed using either Graphpad Prism (San Diego, CA, USA) or Microsoft Office 2013 Excel. Data are presented as Mean ± SEM of all tested groups. The Student’s t-test for independent samples was used to determine the significance of the differences between tested groups.

## Abbreviations

H-ARS	Hematopoietic acute radiation syndrome
IGF-1	Insulin-like Growth Factor-1
IR	Insulin receptor
Ptx3 Slc38a1	Pentraxin 3 Sodium-coupled neutral amino acid transporter 1
Cdkn1a	Cyclin Dependent Kinase inhibitor 1A
NADH	Nicotinamide Adenine Dinucleotide Hydride
PTEN	Phosphatase and tensin homolog
ATP	Adenosine Triphosphate
Nrf2	Nuclear factor erythroid 2–related factor 2
Ucp2	Uncoupling protein 2
ELISA	Enzyme-linked immunosorbent assay
GAPDH	Glyceraldehyde 3-phosphate dehydrogenase
